# A case of muscular bridge resulting in myocardial infraction following heavy effort: a case report

**DOI:** 10.1186/1757-1626-2-135

**Published:** 2009-02-10

**Authors:** Ibrahim Halil Kurt

**Affiliations:** 1 Adana Numune Education and Research Hospital, Department of Cardiology, Adana, Turkey

## Abstract

Muscular bridge (MB) is transient systolic coronary blockage occurring due to exposure of a portion of epicardial coronary arteries to compression during systole as a result of tunneling into the myocardium. Although rare, these patients may develop angina pectoris, severe arrhythmia and myocardial infraction (MI). A 30-year-old male patient presented to the emergency with severe pain with an onset at the front part of the chest followed by spreading to the back and arms, during a football match. The investigations performed revealed anterior wall infraction and thus thrombolytic treatment was administered. Patient's history was normal except for smoking. The patient was detected to play football occasionally since his childhood; however, we learnt that he had started playing without warm-up exercises at the last football match. Coronary angiography detected a lesion with an onset in the left anterior descending artery following the 1st diagonal and extending to the 2nd diagonal and exhibiting a significant contraction during systole. The patient was considered to have myocardial infraction secondary to myocardial bridge. Sudden deaths frequently occur in competitive sports requiring heavy effort.

## Introduction

Myocardial bridge is the most common congenital abnormality of coronary artery disease. It is mainly localized in the proximal part of the left anterior descending artery. Although it leads to angina pectoris, it is usually of benign nature; however, it has been associated with severe ventricular arrhythmias and myocardial infraction [1]. They have a silent clinical course since they are mostly asymptomatic. Post-mortem investigations and IVUS studies reveal a MB rate up to 80% [2]; however it is mostly demonstrated on the investigations performed upon onset of symptoms. In this report, a 30 year old male patient was presented, who experienced anterior MI secondary to MB following severe chest pain with an onset during a compelling football match played without a warm-up exercise period. Although myocardial bridges are asymptomatic, they may still cause sudden death and myocardial infraction during heavy effort.

## Case presentation

**Figure 1:**
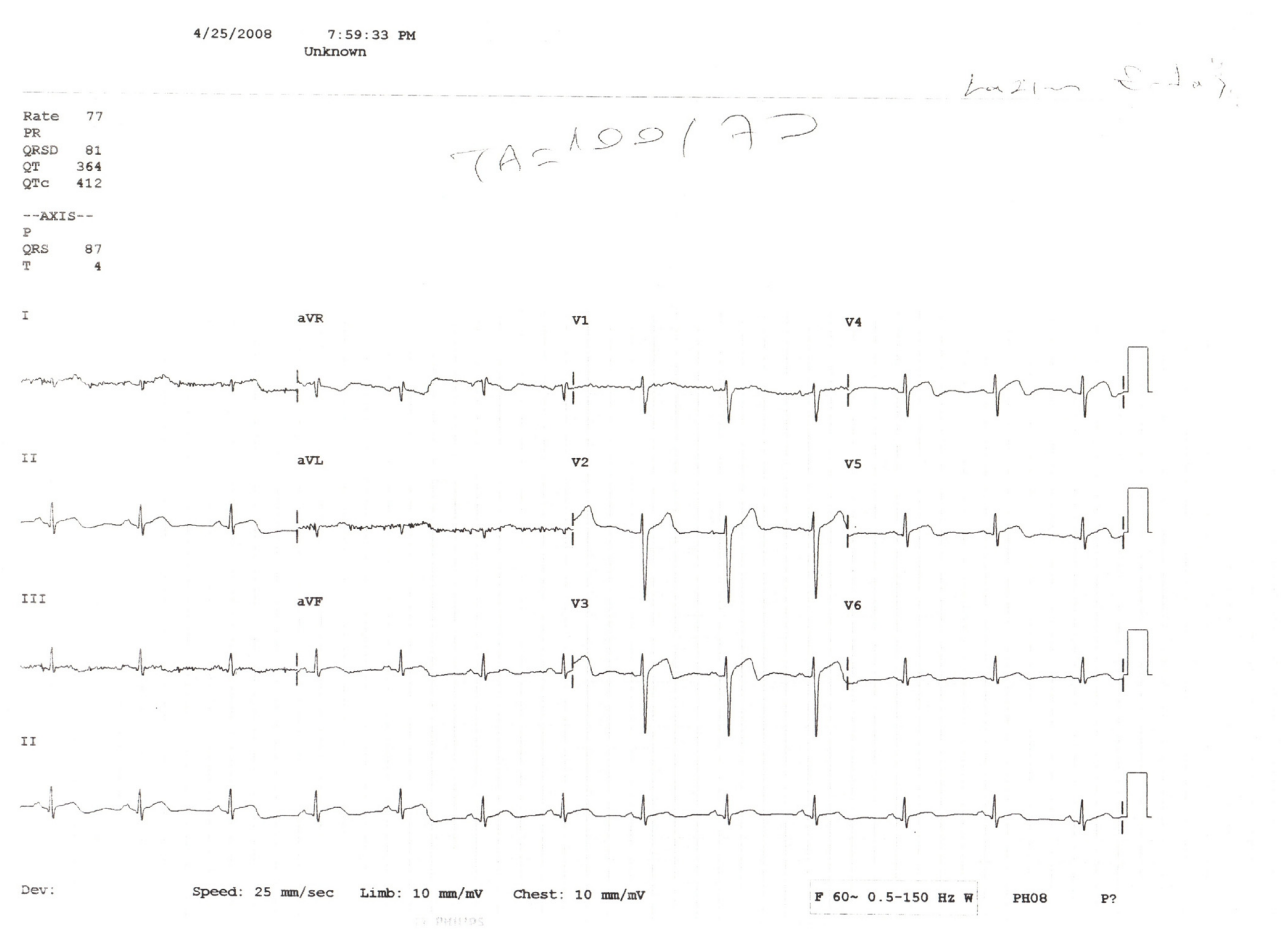
**Electrocardiography reveals ST segment elevation and T negativeness on leads (V2-5)**.

**Figure 2:**
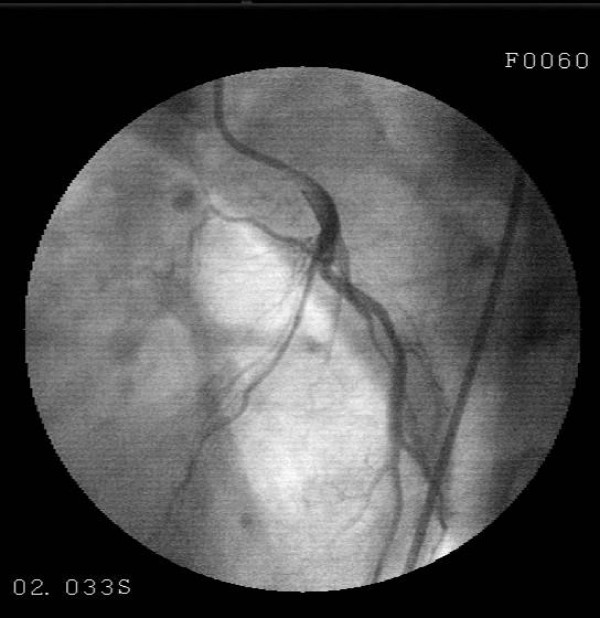
**Coronary angiography performed at left anterior oblique projection shows narrowing in the middle LAD during systole**.

**Figure 3:**
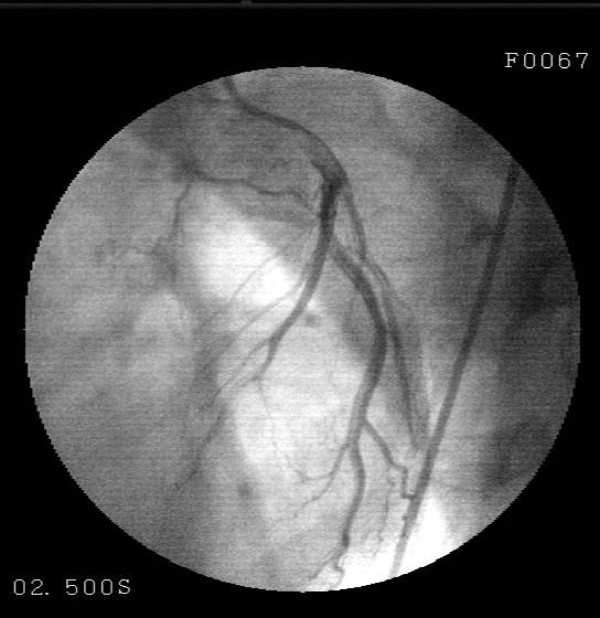
**Coronary angiography performed at left anterior oblique projection shows that the middle LAD returns to normal during diastole**.

**Figure 4:**
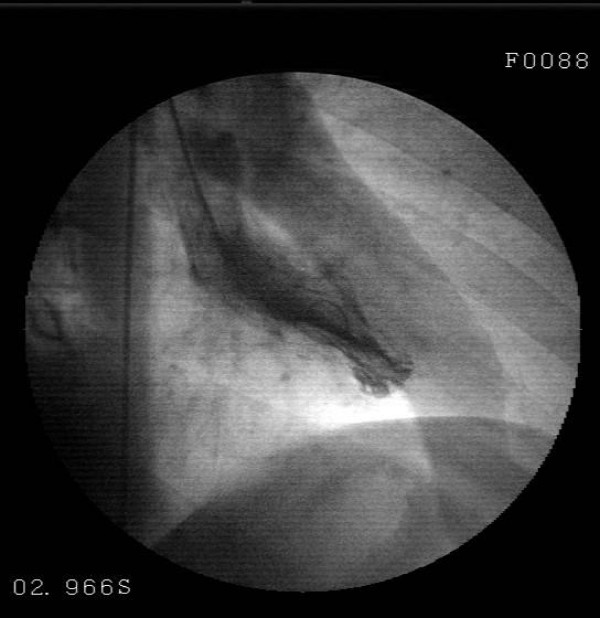
**Ventriculography performed on LAO position reveals minor aneurysm in the apical region**.

A 30-year-old male patient presented to the emergency with severe pain with an onset at the front part of the chest followed by spreading to the back and arms, nausea and excessive sweating developing during a football match. The investigations performed revealed anterior wall infraction and thus he was admitted to coronary intensive care unit. Thrombolytic treatment (streptokinase, 1.5 million IU/1 hour) and metoprolol 50 mg 1 × 1 and ASA 300 mg 1 × 1 were administered. The patient, who recovered from chest pain following thrombolytic treatment, was referred to our center to undergo coronary angiography at Day 3. Patient's history was normal except for smoking (1 packet/day/10 years). The patient was detected to play football occasionally since his childhood; however, we learnt that he had started playing without warm-up exercises at the last football match. His family history included no risk factors regarding coronary artery disease. On physical examination, arterial blood pressure was 120/70 mmHg, pulse was 88 beats/min and the other findings were normal. Electrocardiography revealed ST segment elevation on anterior leads (V2-5) and T negativeness (Figure 1). Troponin I was measured to be 4.54 ng/mL (0.0 – 0.04), blood glucose and lipid panel were within normal limits. Echocardiography detected minor aneurysm in the left ventricular apex. Coronary angiography detected a lesion with an onset in the left anterior descending artery following the 1st diagonal and extending to the 2nd diagonal and exhibiting a significant contraction during systole, which returned to normal during diastole (Figure 2, 3). The right and circumflex artery was normal and ventriculography revealed normal findings except for mild dyskinesia in the apex (Figure 4). Sports requiring heavy effort were prohibited and the patient was discharged with prescriptions of acetyl salicylic acid 300 mg 1 × 1, metoprolol 50 mg 1 × 1. At the follow-up visit taking place 3 months later, he had no complaint.

## Discussion

Muscular bridge is transient systolic coronary blockage occurring due to exposure of a portion of epicardial coronary arteries to compression during systole as a result of tunneling into the myocardium. Myocardial bridge is reported at a rate of 0.5–12% on angiographic series [3,4]. Most of the patients are young males [5]. It is mostly located in the middle LAD and has a length ranging between 1 to 5 cm [6]. Mostly, it is clinically asymptomatic and most of the exercise tests reveal no finding [7]. In cases with myocardial bridge, myocardial infraction is presented in the form of case reports; in most of these cases, the pain occurs during exercise [8,9]. Excessive physical and mental tension is suggested to increase the duration of vasospasm as a result of systolic compression, thereby leading to MI [10]. In addition, systolic compression is suggested to lead to predisposition to thrombus by resulting in endothelium injury [11].

Tachycardia is suggested to induce MI in cases with MB [2]. The IVUS studies usually reveal atherosclerotic plaque in the proximal segment of MB [12]. These plaques are suggested to cause MI by cracking due to mechanic overload [3,9]. Although rare, cases of MI secondary to MB following heavy exercise were reported [13]. Formation of thrombus in the coronary artery was observed in cases of MI secondary to MB following excessive compelling [7]. In our case, although LAD had a MB in a long segment, there was injury in a very small segment of the myocardium; this possibly resulted from the contribution of ischemic preconditioning as well as administration of thrombolytic treatment [14]. Although the rate of MB cases is high, treatment is administered based on symptoms. Exercise test, myocardial perfusion scintigraphy, magnetic resonance and coronary flow reserve Doppler techniques are used to detect ischemia. However, in cases with MB, perfusion scintigraphy performed with vasodilating agents such as dipyridamole is reported to yield false results due to steal syndrome [15]. For MB cases with findings of ischemia, there are therapeutical choices of coronary stent, coronary bypass and myotomy as well as medical treatment. Despite therapeutical choices, percutaneous and surgical treatment does not achieve much success. Therefore, beta-blockers and Ca++ antagonists are usually preferred as medical treatment. In such cases, use of beta-blockers is recommended, particularly in case of heavy exercise. In such cases, acetyl salicylic acid may be added to treatment for prophylactic purposes. The myocardial (MIBI) perfusion scintigraphy performed after 3 months while the patient was on beta-blocker revealed no ischemic findings.

## Conclusion

Although the incidence of MB is very high in the society, it is not considered very important since MB does not cause serious complaints. Although rare, mortal outcomes such as MI and sudden death may be observed in some cases. Sudden deaths frequently occur in competitive sports requiring heavy effort.

## Consent

Written informed consent was obtained from the patient for publication of this case report and accompanying images. A copy of the written consent is available for review by the Editor-in-Chief of this journal.

## Competing interests

The author declares that they have no competing interests.
